# Recent Progress in Research of Additive Manufacturing for Polymers

**DOI:** 10.3390/polym14112267

**Published:** 2022-06-02

**Authors:** Swee Leong Sing, Wai Yee Yeong

**Affiliations:** 1 Department of Mechanical Engineering, National University of Singapore, Singapore 117575, Singapore; 2 Singapore Centre for 3D Printing, School of Mechanical & Aerospace Engineering, Nanyang Technological University, Singapore 639798, Singapore; wyyeong@ntu.edu.sg

Additive manufacturing (AM) methods have grown and evolved rapidly in recent years. AM for polymers is particularly exciting and has great potential in transformative and translational research in many fields, such as biomedical [1,2,3], aerospace [4,5], and electronics [6,7]. Current methods for polymer AM include material extrusion, material jetting, vat photopolymerization, and powder bed fusion. As these techniques matured and developed, more functionalities have been added to AM parts. Such functionalities include multi-material fabrication [8,9,10] and integration with artificial intelligence [11]. These have resulted in polymer AM to evolve from a rapid prototyping tool to actual manufacturing solution. 

In this special issue, state-of-the art research and review articles are collected. They focus on the process–structure–properties relationships in polymer AM. In total, one review and nine original research articles are included. Gülcan et al. provided a comprehensive review on the material jetting technique for polymer AM by analyzing the effect of the critical process parameters and providing benchmarking with other manufacturing processes [12]. In their research, Nagarajan et al. investigated the use of polymer composites that contain ferromagnetic fillers for applications in electronic and electrical devices. These composites were processed using material jetting and alignment of the fillers was achieved using magnetic field [13]. Wu et al. also used material jetting to produce novel composite materials that are multi-material [14]. Udroiu studied the use of material jetting produced surfaces for aerodynamic models [15]. Samat et al. evaluated the mechanical and in vitro properties of material extruded thermoplastic polyurethane and polylactic acid blend for tracheal tissue engineering [16]. Zhang et al. also used material extrusion of blends for their experiments. They studied biodegradable polyesters and adjusted the blend compositions to tailor the mechanical performance [17]. Catana et al. studied the bending resistance of polylactic acids and compared them to the simulations. They found that the AM parts deviated from simulations due to fluctuations in process parameters [18]. Jiang and Drummer studied the effect of curing strategy on the part accuracy produced by vat photopolymerization [19]. Gueche et al. investigated the feasibility of using di-carboxylic acids to produce solid oral forms with copovidone and ibuprofen using powder bed fusion [20]. Finally, Schlicht et al. developed new scanning strategies using quasi-simultaneous exposure of fractal scan paths for powder bed fusion of polymers that can reduce the energy consumption of the process [21]. 
